# Analysis of Ambulatory Proctologic Surgery for Simple Anal Fistulas in Terms of Recovery, Complications, Recurrence, and Cost

**DOI:** 10.7759/cureus.42110

**Published:** 2023-07-19

**Authors:** Hakan Demir, Recayi Capoglu, Merve Yigit, Tarik Harmantepe, Emre Gonullu, Kerem Karaman

**Affiliations:** 1 Department of General Surgery, Sakarya University Research and Education Hospital, Sakarya, TUR; 2 Department of General Surgery, Sakarya University Training and Research Hospital, Sakarya, TUR; 3 Department of Gastrointestinal Surgery, Sakarya University Training and Research Hospital, Sakarya, TUR

**Keywords:** cost, ambulatory surgery, loose seton, fistulotomy, anal fistula

## Abstract

Background

Ambulatory anorectal surgeries have increased in the last few years. This clinical study aimed to compare general operating room conditions with outpatient procedures for simple anal fistulas in terms of healing success, recurrence, cost, complications, and sustainability.

Methodology

Only primary fistulotomy and seton application for simple anal fistulas were retrospectively analyzed.

Results

Two-hundred fifty patients (73.7%) were male, and 89 (26.3%) were female. Sixty patients (17.7%) were treated in the operating room, and 279 (82.3%) were treated in the outpatient clinic conditions. Of the ambulatory surgeries, 160 patients underwent fistulotomy and 119 patients loose seton. On the other hand, 34 patients underwent fistulotomy and 26 patients loose seton in operating room conditions. No significant difference was found between the groups according to the distribution of age, gender, complications, and recurrence (*P *> 0.05). Cost-effectiveness assessment according to the place (ambulatory/operating room) and type of operation (fistulotomy/loose seton) reveals that ambulatory surgery provides significantly more savings (*P *< 0.001).

Conclusions

For simple anal fistulas, ambulatory anorectal surgery is a safe approach that can be performed at a lower cost than operating room conditions.

## Introduction

Proctological local interventions have increased in the last six decades. Since the creation of the first modern outpatient surgery program, it has been followed by two major centers at the University of California (Los Angeles) and Phoenix (Arizona). The positive results of these centers set an example for centers in other countries [1].

In the United States, 16% of surgeries were performed on an outpatient basis in 1980, while it increased to 67% in 2000. Currently, approximately 50% of all proctologic surgeries performed in the United Kingdom are outpatients without the need for hospitalization [2,3].

Anorectal diseases such as simple anal fistulas (less than 4 cm from the anus), chronic anal fissures that do not respond to medical treatment, grades II and III hemorrhoids, pilonidal sinus, anal abscesses, and small neoplasms are suitable for outpatient surgery [4].

In outpatient proctological surgeries, the treatment of fistulas is most challenging for surgeons. As the recovery time, recurrence, and incontinence probability are higher, patient satisfaction decreases. Treatment of grade II hemorrhoids and chronic anal fissures with surgery or new technological instruments are more successful in terms of recovery and patient satisfaction.

The primary aim of this retrospective clinical study was to compare general operating room conditions with outpatient procedures for simple anal fistulas in terms of healing success, recurrence, cost, complications, and sustainability. Our second aim is to determine how many conventional radiological additional tests are needed by outpatients and to determine the burden of this on radiology, operation waiting time, and cost.

## Materials and methods

After the approval of the Sakarya University Ethics Committee, patients who were operated on for simple anal fistula between January 2018 and July 2022 were retrospectively analyzed. A single surgeon operated on study patients. Outpatients suitable for the day surgery program, predominantly the American Society of Anesthesiologists scores (ASA) I and II were preferred. Patients who were within 30 to 60 minutes of reaching the hospital in an emergency and were ready to undergo local surgery in psychosocial terms were included.

Nonsurgical options for complicated anal fistulas, grades III and IV hemorrhoids, chronic anal fissures, and grades I and II hemorrhoids, which require more general operating room conditions were excluded from this study. For the objectivity of the study, only simple anal fistulas were included.

Simple intersphincteric and transsphincteric fistulas with a distance of less than 4 cm from the anus in both the outpatient clinic and the operating room were included in the study. According to the lithotomy position, female patients, especially anterior fistulas and horseshoe, complicated grades III and IV fistulas, were excluded from the study. In the digital examination performed in the General Surgery Outpatient Clinic - depending on the fibrosis status of the anal canal sphincters - it was decided to perform primary fistulotomy or loose seton. Primary fistulotomy was performed for simple anal fistulas (usually intersphincteric fistulas) with sphincter fibrosis, 2 cm or less from the anus. Loose seton was applied to those with a diameter of up to 4 cm (i.e., intersphincteric and low transsphincteric fistulas below 1/3 of the external sphincter) and those with insufficient sphincter fibrosis.

We used a 6 cm length part of the 2.67 mm (0.8) CH minivac drain (Bıçakçılar, Turkey) for the loose seton. Secondary fistulotomy was performed after the loose seton procedure was retained for at least 3 months or more.

After both primary fistulotomy and seton application, the controls of the patients were performed at one, two, and three-month intervals, and the data were recorded. Those who spent the first three months without any problems were called for six-month controls. Those who had an uneventful three months in primary fistulotomy were considered completely healed. Setons were opened for at least three months, and in the following months, depending on the healing of the fistula canal and the formation of fibrosis, the seton was removed by opening the canal with a secondary fistulotomy. If the wound healing was complete at the sixth-month follow-up, it was considered to be completely healed.

Endorectal ultrasound and pelvic magnetic resonance imaging (MRI) were requested for patients whose internal opening could not be determined and who were found to have complex fistulas. Thus, their operations were planned in the general operating room.

Ultrasound and MRI procedures, surgery prices of the patients, and the savings of the institution (i.e., profitability ratios) of the patients who were operated out of day or in the general operating room were calculated by considering the price application notification of the Ministry of Health of the Republic of Turkey. It is stated in American dollars based on international annual exchange rates.

Due to variations in patients' pain thresholds and the subjective nature of the results, evaluation using the Visual Analog Scale (VAS) pain scale was not conducted. Patient satisfaction is also subjective. A separate scale was not prepared for patient satisfaction.

The recurrence, complication, success rates, and waiting times of the patients who were operated on in the operating room or outpatient were compared.

Statistical analysis

Data analysis was done in IBM SPSS Statistics, Version 25 (IBM Corp., Armonk, NY, USA) package program. The Kolmogorov-Smirnov and Levene’s tests were, respectively, used to investigate whether the assumptions of normal distribution and homogeneity of variances were met. Categorical data were expressed as numbers (*n*) and percentages (%), while quantitative data were given as mean ± standard deviation (SD) and median (min-max). While the mean differences between groups were compared by student’s *t*-test, otherwise Mann-Whitney U test was applied for comparisons of the not normally distributed data. Qualitative data were analyzed by *χ*^2^ or Fisher’s exact test, where appropriate. Recurrence-free survival (RFS) was computed by the method of Kaplan-Meier survival analyses, and categorical variables were compared by the log-rank test. Crude survival (success) ratios and mean expected duration of life with 95% confidence intervals (CIs) for each subgroup were also calculated. A *P*-value <0.05 was considered statistically significant.

## Results

Table 1 presents a comparison of demographic and clinical characteristics between the cases treated in the operating room and those treated in the outpatient clinic.

**Table 1:** Demographic and clinical characteristics of the cases according to the groups whether the patients were intervened in the operating room or the outpatient clinic. Descriptive statistics. *Mean ± standard deviation. **Median (minimum-maximum). ^†^Student’s *t*-test. ^‡^Yates chi-square (*χ*^2^) test. ^¶^Mann-Whitney U test. ^§^ *χ*^2^ test. ^¥^Fisher's exact probability test. N/A, no evaluation

	Total (*n *= 339)	Operating room (*n *= 60, 17.7%)	Polyclinic (*n *= 279, 82.3%)	*P*-value
Age (Years)*	42.6 ± 12.7	42.3 ± 12.6	42.7 ± 12.8	0.821^†^
Gender				
Male	250 (73.7%)	48 (80%)	202 (72.4%)	0.293^‡^
Female	89 (26.3%)	12 (20%)	77 (27.6%)	
Disease duration (month)**	12 (1-360)	24 (3-360)	12 (1-360)	0.012^¶^
Fistula length*	3.2 ± 1.3	3.7 ± 1.5	3.0 ± 1.2	<0.001^†^
Fistula type				
Intersphincteric	151 (44.5%)	14 (23.3%)	137 (49.1%)	<0.001^§^
Transsphincteric	188 (55.5%)	46 (76.7%)	142 (50.9%)	
Operation type				
Fistulotomy	194 (57.2%)	34 (56.7%)	160 (57.3%)	0.923^§^
Seton	145 (42.8%)	26 (43.3%)	119 (42.7%)	
Fecal soiling	6 (1.8%)	3 (5.0%)	3 (1.1%)	0.071^¥^
Hospitalization	58 (17.1%)	55 (91.7%)	3 (1.1%)	N/A
Hospitalization period (day)^**^	2 (2-7)	2 (2-7)	3 (2-4)	N/A
Bleeding	3 (0.9%)	0 (0.0%)	3 (1.1%)	>0.999^¥^
Hypotension	8 (2.4%)	1 (1.7%)	7 (2.5%)	>0.999^¥^
Disease outcome				
Successful	313 (92.3%)	53 (%88.3%)	260 (93.2%)	0.191^¥^
Recurrence	26 (7.7%)	7 (11.7%)	19 (6.8%)	
Follow-up (month)^**^	30 (2-54)	17 (2-53)	31 (2-54)	N/A

In this study, data from 339 cases, ranging in age from 17 to 76 years, were evaluated. The mean age of the cases was 42.6 ± 12.7 years, 250 patients (73.7%) were male, and 89 (26.3%) were female. While 60 patients (17.7%) were treated in the operating room, 279 (82.3%) were treated in outpatient clinic conditions. There was no significant difference between the groups in terms of mean age, distribution of males and females, type of surgery, fecal contamination, bleeding, and hypotension (*P *> 0.05).

The median disease duration and mean fistula length of the group treated in the outpatient clinic were significantly less than the group treated in the operating room (*P *= 0.012 and *P *< 0.001). Intersphincteric fistula type was significantly more frequent in the group treated in the outpatient clinic, while transsphincteric fistula was used less frequently (*P *< 0.001).

**Figure 1:**
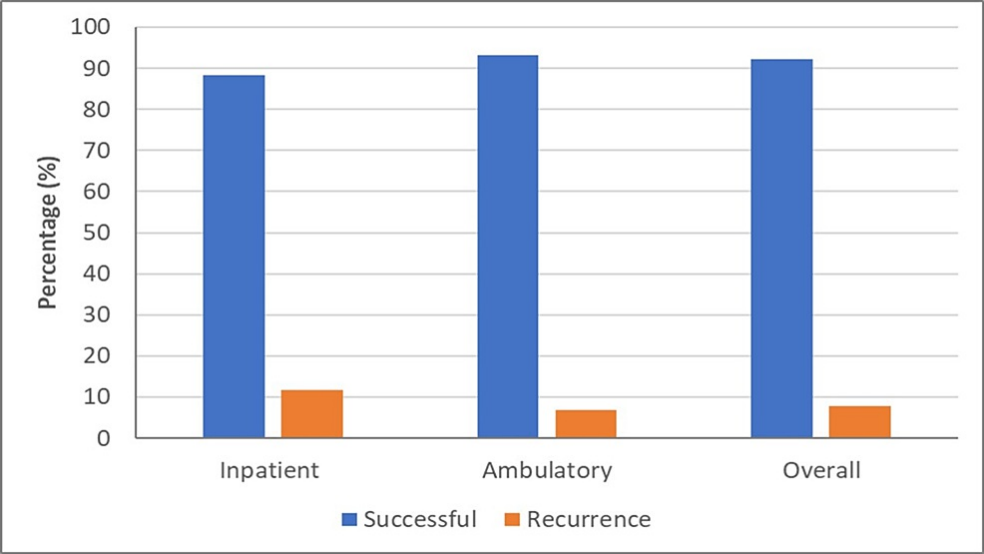
Success and recurrence rates according to inpatient and ambulatory settings.

Although the success rate was higher and the recurrence rate was lower in the group treated in the outpatient clinic compared to the group treated in the operating room, the difference was not statistically significant (Figure 1).

Table 2 shows the comparisons made in terms of the need for radiological examination, the year of the procedure, and the cost of the procedure, according to the group that was intervened in the operating room and the group that was intervened in the outpatient clinic. In addition, the need for ultrasound was found to be statistically significantly less in the group treated in the outpatient clinic compared to the group treated in the operating room (*P *= 0.008). Although the MRI requirement was less in the group treated in the outpatient clinic compared to the group treated in the operating room, the difference was not statistically significant (*P *= 0.312).

**Table 2:** The need for radiological examination, the year of the procedure, and the cost of the procedure according to the groups. ^†^Yates chi-square test. ^‡^Mann-Whitney U test. N/A, no evaluation

	Total (*n *= 339)	Operating room (*n *= 60)	Polyclinic (*n *= 279)	*P*-value
Ultrasound requirement				
No	280 (82.6%)	42 (70.0%)	238 (85.3%)	0.008^†^
Yes	59 (17.4%)	18 (30.0%)	41 (14.7%)	
Ultrasound cost				
World currency (USD)	0.34 ± 0.75	0.56 ± 0.88	0.29 ± 0.71	0.009^‡^
MRI requirement				
No	252 (74.3%)	41 (68.3%)	211 (75.6%)	0.312^†^
Yes	87 (25.7%)	19 (31.7%)	68 (24.4%)	
MRI cost				
World currency (USD)	2.58 ± 4.44	3.11 ± 4.68	2.46 ± 4.38	0.390^‡^
Transaction year				
2018	16 (4.7%)	6 (10.0%)	10 (3.6%)	N/A
2019	141 (41.6%)	12 (20.0%)	129 (46.2%)	
2020	66 (19.5%)	11 (18.3%)	55 (19.7%)	
2021	79 (23.3%)	22 (36.7%)	57 (20.4%)	
2022	37 (10.9%)	9 (15.0%)	28 (10.0%)	
Overall cost				
World currency (USD)	138.0 ± 19.8	135.1 ± 27.3	138.7 ± 17.8	0.081^‡^
Institution savings				
World currency (USD)	114.7 ± 26.9	67.6 ± 13.6	124.8 ± 16.1	<0.001^‡^

The amount spent for ultrasound was statistically significantly lower in the group treated in the outpatient clinic compared to the group treated in the operating room (*P *= 0.002 and *P *= 0.009).

The costs of transactions were statistically similar between the group treated in the operating room and the group treated in the outpatient clinic (*P *= 0.081).

It was observed that the amount of savings achieved by the institution in terms of foreign currency (USD) was statistically significantly higher in the group treated in the outpatient clinic compared to the group treated in the operating room (*P *< 0.001).

**Figure 2:**
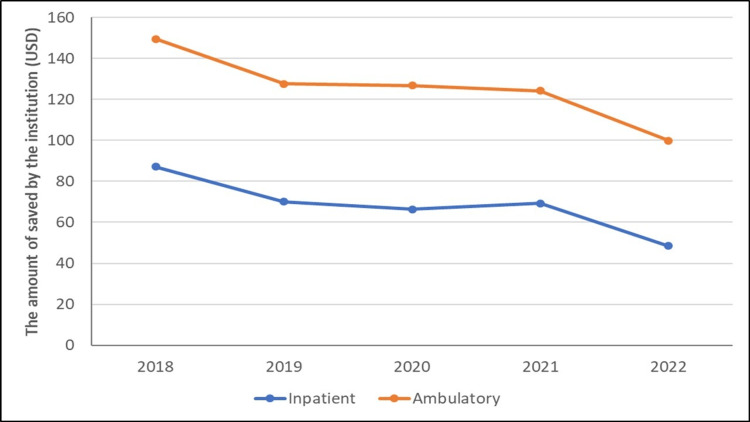
The amount of savings achieved by the institution (USD).

In Figure 2, the amount of savings achieved by the institution in foreign currency (USD) by the group treated in the operating room and the outpatient clinic is shown as a line plot.

In Table 3, the evaluations made in terms of cost-effectiveness according to the place and type of operation are given. It was observed that the amount of savings achieved by the institution in terms of foreign currency (USD) was statistically significantly higher in the group treated in the outpatient clinic compared to the group treated in the operating room (*P *< 0.001). Among the cases in which seton was applied, it was observed that the amount of savings achieved by the institution in terms of foreign currency (USD) was significantly higher in the group treated in the outpatient clinic compared to the group treated in the operating room (*P *< 0.001). Among the cases treated in the operating room, the savings achieved by the institution in terms of foreign currency (USD) were found to be statistically similar (*P *= 0.223 and *P *= 0.685). Among the cases treated in the outpatient clinic, the amount of savings achieved by the institution in terms of foreign currency (USD) was found to be similar between the group that underwent fistulotomy and the group that underwent seton (*P *= 0.099 and *P *= 0.111). Finally, in all cases, the amount of savings achieved by the institution in terms of foreign currency (USD) was found to be statistically similar between the group that underwent fistulotomy and the group that underwent seton (*P *= 0.106 and *P *= 0.257).

**Table 3:** Cost-effectiveness assessment according to the place and type of operation where the operation was performed. Descriptive statistics: mean ± standard deviation. ^†^Comparisons between the operating room and polyclinic within the types of operations. ^‡^Comparisons between operation types within the operating room and outpatient clinic groups. ^¶^Mann-Whitney U test.

	Operating room	Polyclinic	*P*-value^†¶^	Total
Institution savings (USD)				
Fistulotomy	66.6 ± 15.2	123.1 ± 15.7	<0.001	113.2 ± 26.5
Seton	68.7 ± 11.6	127.0 ± 16.3	<0.001	116.6 ± 27.3
*P*-value^‡¶^	0.685	0.111		0.257

Table 4 shows the RFS results according to the group treated in the operating room and the group treated in the outpatient clinic. The overall survival (success) rate was 92.3% among all cases, and it was observed that the cases could live without recurrence for an average of 50 months (95% CI 48.5-51.5). The crude survival (success) rate of the group treated in the operating room was 88.3%, with an expected mean RFS of 46.4 months (95% CI 41.8-51.0). The overall survival rate of the intervention group in the outpatient setting was 93.2%, with an expected mean RFS of 50.5 months (95% CI 48.9-52). There was no statistically significant difference between the groups in terms of RFS (*P *= 0.154).

**Table 4:** Recurrence-free survival results according to the group treated in the operating room and the group treated in the outpatient clinic. *Results: mean recurrence-free survival and 95% confidence interval. **Cases in which USG or MRI was undesirable during the intervention. USG, ultrasound; MRI, magnetic resonance imaging

	n	Recurrence	Overall survival rate (%)	Disease-free survival (month)*	Log-rank	*P*-value
Operation site					2.028	0.154
Operating room	60	7	88.3	46.4 (41.8-51.0)		
Polyclinic	279	19	93.2	50.5 (48.9-52.0)		
Operation site**					1.567	0.211
Operating room	30	2	93.3	49.4 (44.6-54.2)		
Polyclinic	188	5	97.3	52.6 (51.4-53.8)		
Total	339	26	92.3	50.0 (48.5-51.5)	-	-

In cases where additional radiological examinations such as ultrasound or MRI were not requested during the intervention, the crude survival (success) rate of the group treated in the operating room was 93.3%, with an expected mean RFS time of 49.4 months (95% CI 44.6-54.2). The crude survival (success) rate of the intervention group in the outpatient setting was 97.3%, with an expected mean RFS of 52.6 months (95% CI 51.4-53.8). There was no statistically significant difference between the groups in terms of RFS (*P *= 0.211).

The mean length of stay of the patient in the outpatient and hospital surgical unit was 3.8 and 324 hours, respectively. Outpatient surgery costs 28.4% of the hospital stay in patients with similar interventions. Surgical treatment of the aforementioned diseases can be done by outpatient treatment of more than 50% of the patients.

Three (1.1%) patients who were operated on in the outpatient clinic were hospitalized due to bleeding. Seven (2.5%) patients were discharged due to the development of hypotension, and their vital signs improved after intervention in the emergency department. Transient fecal contamination developed in the follow-up of six patients, three (1.1%) in the outpatient group and three (5%) in the operating room group. In the sixth month follow-up, fecal contamination improved. In face-to-face interviews with patients, approximately 89% stated that they were satisfied with the outpatient surgery. The most frequently reported causes were related to seton intolerance.

## Discussion

As a result of the experience gained, since 90% of proctological diseases can be performed with local anesthesia on an outpatient basis, it has been revealed that patients with anal diseases do not need to stay in the hospital for days, and recovery follow-up can be performed on an outpatient basis [5].

Today, with the development of local anesthetics, more comfortable surgeries can be performed without the need to combine them with sedation agents. With the development of new anesthetic agents and outpatient surgical techniques that give confidence to both the patient and the surgeon in daily practices, the length of hospital stay and costs have decreased [6].

Large hospitals are structures where expensive equipment is combined and surgical procedures are performed with great safety and comfort. As a result, the preference for surgeons has shifted to large hospitals, causing long waiting lists for hospitalization, a shortage of beds for major surgeries, and high bed/day costs. In the study by Nahas et al. [7], patients waited on average for 18 months, whereas in our case series, patients in the general operating room had to wait at least six months before undergoing surgery.

The classification of anal fistula, which was explained for the first time by Parks, was revised over time. Because the Parks classification could not fully explain simple and complicated fistulas. In our study, the revised classification of Garg [8] was used.

As stated in the guidelines, up to 30% of the external sphincter muscle can be cut without compromising fecal continence [9,10,11]. Canal resection is often not required in simple fistulas. Secondary fistulotomy may be preferred after primary fistulotomy or seton materials are used in the treatment after the secondary canals are destroyed within a certain period of time.

Although it is accepted as the gold standard in loose seton complex fistulas, as reported by Kelly et al. [12], there are few studies on its use in simple fistulas, the success rates rise to 73%, and the patient tolerance reaches 95%.

In the present study, In secondary fistulotomy after primary fistulotomy and seton application, the success rate in the group that was intervened in the operating room was 88.3%, and the success rate of the group that was intervened in the outpatient clinic was 93.2%.

As reported in the study by Cirocco and Reilly [13], it was observed that the outer and inner orifices of our cases followed Goodsall's rule in 54% of the cases. This finding suggests that Goodsall's rules may have lost some of their former significance.

We observed that the amount of savings obtained by the institution in terms of foreign currency (USD) was significantly higher in the group treated in the outpatient clinic compared to the group treated in the operating room among the cases who underwent seton with primary fistulotomy (*P *< 0.001). There was a significant difference between the groups that were treated in the operating room and the groups that were treated in the outpatient clinic in terms of institutional profitability. According to the price application communiqué of the Ministry of Health of the Republic of Turkey, the price of anal fistula surgery for five years (taking into account the average international exchange rate) is 138.0 ± 19.8 USD. The average institutional profitability per case is 67.6 ± 13.6 USD in cases performed in the operating room and 124.8 ± 16.1 USD in cases performed in the outpatient clinic.

Although the price policies for the surgery are different for each country, in the study by Nahas et al. [7], they reported that the cost of surgery for a one-day hospitalized patient is 570.00 USD and 390.00 USD for an outpatient.

Fisher et al. [14], in a comparison of the fibrin plug and endorectal advancement flap of anal fistulas, reported that the application of fibrin plugs resulted in a cost saving of 2518 Euros compared to the application of the endorectal flap. It should be considered that this may result in cost savings for simple outpatient anal fistulas. 

We could not find any study in the literature about how the conventional radiological system is used for the classification of anal fistulas. Many surgeons believe that the distance from the external opening to the anal border leads to fistula complexity and, therefore, predicts the need for additional imaging. However, there is no evidence to support this. Preoperative imaging is recommended for fistulas with external openings >1 cm from the anal verge [15].

In our study, 17.4% (59/339) of the cases where internal orifices and fistula types could not be identified required ultrasound and 25.7% (87/339) needed MRI. The average cost of ultrasound by year was 0.34 ± 0.75 USD, and the cost of MRI was 2.58 ± 4.44 USD. In the other cases, without any radiological procedure, both the type of fistula and the operation could be performed with local anesthesia in the outpatient clinic. This means that when determining the fistula type under local anesthesia, we can state how unnecessary ultrasonography and MRI are requested, and this increases the occupation of the relevant departments. 

In the study by Sobrado et al. [16], they explained that the most common complication in all proctologic cases was severe pain (16.1%). They stated that five patients (1.5%) required hospitalization due to postoperative complications. In the series by Kulkarni et al. [17], the patient satisfaction rate was 93%, and they explained that 6.7% of the patients had urinary retention.

Mulita et al. [18] reported that the most significant cause of regional and deep surgical site infections was the presence of living organisms in the area before the wound was closed.

In the patient group, we operated on, for patients with a short fistula tract and regional sepsis, a secondary fistulotomy was performed following seton drainage to control the sepsis. This was done after waiting for at least 3 months or more, and only after ensuring complete healing. Therefore, in a few patients, delayed wound healing and lack of proper drainage were observed, which were attributed to the formation of false tracts.

In our cases, three (1.1%) patients required hospitalization due to bleeding. In three (1.1%) patients, hypotension developed, which improved with medical treatment. Urinary retention was not detected in any patient in the outpatient and general operating room groups. Severe pain was observed in 4% (15/339) of the patients in both groups.

The limitation of the study is that pressures were not measured with an anal manometer in patients with fecal incontinence.

## Conclusions

Outpatient anorectal surgery is a safe and cost-effective approach, especially in the treatment of simple anal fistulas. Increasing the savings rate of hospitals will make a significant contribution to the national economy. In order to support daily anorectal surgery, it is necessary to control pain very well. It can be concluded that local anesthesia is a safe and advantageous technique in the outpatient treatment of simple anal fistulas.
